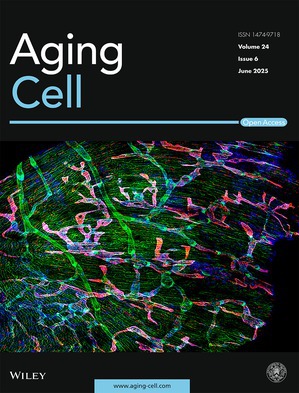# Featured Cover

**DOI:** 10.1111/acel.70128

**Published:** 2025-06-10

**Authors:** Kangsan Roh, Haobo Li, Rebecca Nicole Freeman, Luca Zazzeron, Ahlim Lee, Charles Zhou, Siman Shen, Peng Xia, Justin Ralph Baldovino Guerra, Cedric Sheffield, Timothy P. Padera, Yirong Zhou, Sekeun Kim, Aaron Aguirre, Nicolas Houstis, Jason D. Roh, Fumito Ichinose, Rajeev Malhotra, Anthony Rosenzweig, James Rhee

## Abstract

Cover legend: The cover image is based on the article *Exercise‐Induced Cardiac Lymphatic Remodeling Mitigates Inflammation in the Aging Heart* by Kangsan Roh et al., https://doi.org/10.1111/acel.70043.